# Structure Optimization of a Grain Impact Piezoelectric Sensor and Its Application for Monitoring Separation Losses on Tangential-Axial Combine Harvesters

**DOI:** 10.3390/s150101496

**Published:** 2015-01-14

**Authors:** Zhenwei Liang, Yaoming Li, Zhan Zhao, Lizhang Xu

**Affiliations:** Key Laboratory of Modern Agricultural Equipment and Technology, Ministry of Education & Jiangsu Province, Jiangsu University, Zhenjiang, Jiangsu 212013, China; E-Mails: liangzhenwei518@126.com (Z.L.); zhaozhan@ujs.edu.cn (Z.Z.); justxlz@ujs.edu.cn (L.X.)

**Keywords:** grain separation losses, monitoring mathematics, structure optimization, field experiment

## Abstract

Grain separation losses is a key parameter to weigh the performance of combine harvesters, and also a dominant factor for automatically adjusting their major working parameters. The traditional separation losses monitoring method mainly rely on manual efforts, which require a high labor intensity. With recent advancements in sensor technology, electronics and computational processing power, this paper presents an indirect method for monitoring grain separation losses in tangential-axial combine harvesters in real-time. Firstly, we developed a mathematical monitoring model based on detailed comparative data analysis of different feeding quantities. Then, we developed a grain impact piezoelectric sensor utilizing a YT-5 piezoelectric ceramic as the sensing element, and a signal process circuit designed according to differences in voltage amplitude and rise time of collision signals. To improve the sensor performance, theoretical analysis was performed from a structural vibration point of view, and the optimal sensor structural has been selected. Grain collide experiments have shown that the sensor performance was greatly improved. Finally, we installed the sensor on a tangential-longitudinal axial combine harvester, and grain separation losses monitoring experiments were carried out in North China, which results have shown that the monitoring method was feasible, and the biggest measurement relative error was 4.63% when harvesting rice.

## 1. Introduction

Combine harvesters have been playing an increasingly important role in modern agricultural production in recent years, and their working process can be divided into the following operations: cutting of the crop and recovering grains from the field; separating grains from the greater crop parts such as straw; separating grains from material-other-than-grain (MOG); and collecting cleaned grains into a tank for temporary storage. Developments in agriculture have brought about greater and more complex harvesters. To increase harvesting efficiency, a combine driver mainly concentrates on the speed of the combine harvester and the height of the header, paying little or no attention to internal processes [1], which would cause unpredictable grain losses in the field working process, and results in a direct loss of income for the farmers.

Grain separation losses are an important parameter to weigh the performance of a combine harvester. The traditional separation losses monitoring method mainly rely on manual labor, using an oil skin to collect all the mixed material at the exhaust port, then filtering out the grains from MOG manually, and proceeding to weigh them and thus calculate the separation losses. This requires such a heavy workload, and is a cumbersome and time-consuming method. With recent advancements in sensors, electronics and computational processing power, automated technologies for combine harvesters have been made possible is part [1–4] and some researchers have proposed many sensors for combine harvesters to extract immediate information from the working process [5], either monitoring the machine settings, the machine load or the field-related parameters [6–10]. To keep combine harvesters working on optimal conditions and grain losses within acceptable limits in a timely way, some scholars have been engaged in recent years in studying grain separation losses' auto-detection technology. The monitoring methods presented in the previous literature were mainly concentrated on three aspects:

(1) Detecting separation losses from the mixture at the exhaust port directly. Some scholars installed a force-electric sensor at the exhaust port, and then calculated the current grain separation losses according to the differences in collision signals. Because the mixed material is discharged from the exhaust port with a relative higher speed and the interactions between grains and MOG, this method could not guarantee that all discharges would generate electrical signals, so the monitoring accuracy could not be guaranteed. Zhang *et al.* used image processing methods to detect separation losses in combine harvesters, however, the corresponding field experiment results indicated that the relative error of image processing methods was too large and therefore limited to laboratory testing [11].
(2) Installation of piezo-electric or acoustical impact sensors at the end of the walker to indicate separation losses by quantifying the grain impacts that occurred in each second. Maertens *et al.* measured separation losses with two impact sensors at the end of the walker and proposed an on-the-go monitoring algorithm to analyze the behavior of the separation processes in combine harvesters [12]. Liu *et al.* proposed a system which installed acoustical grain impact sensors on the harvester to monitor the separation losses in real-time. However, it was very difficult to distinguish grain and MOG collisions from machine noise; the acoustic sensors need to have high resolution, and the signal-conditioning circuitry is complex [13]. Apparently, the grain impact sensor installed at the end of the walker was just a loss indicator, no absolute grain loss measurements were achieved and time-delays are unavoidable.
(3) Selection of relevant input variables to predict grain losses. Schneider developed an exponential function for predicting separation losses based on measurement of crop throughput [14]. Tang *et al.* established a threshing-separating matrix equation to calculate grain separation losses on a tangential-longitudinal-axial combine harvester. However, field experiments showed that the relative measurement error was nearly 6% compared with manual measurements [15]. To date, no recent literature has been found that discusses a method to monitor grain separation losses in real time with relative higher accuracy.

Measurement accuracy of a grain impact sensor is a key factor for monitoring grain separation losses. In recent years, some researchers have engaged in the study of grain losses auto-detection technology and many advanced combine harvesters have already installed grain impact sensors to monitor grain losses by quantifying grain impacts that occurred each second [1–5,16]. W. Eldredge fixed a piezoelectric crystal on the cleaning sieve, and then the electric signal caused by grain collisions was amplified and transmitted to a counting device. However, this monitoring method has some disadvantages, such as small detection range, strong signal interference and fragile piezoelectric ceramics due to oscillating sieving [17]. Osselaere put the sensor into a sealed enclosure to abate the external interference on static performance, but the dynamic performance of the sensor was not found to be improved, and the influence of vibration and ground bumps still cannot be controlled during harvesting [18]. Li *et al.* provided some improvements to improve the sensitivity and the dynamic characteristic of the grain impact sensor, however, the sensitivity distribution on the plate surface is still uneven, its accuracy was still relatively low and furthermore, the proposed measures were not applied in the field [19]. Li and Jie proposed a grain loss detection method based on a virtual test system, but the method is still under research [20]. Ni *et al.* designed a piezoelectric crystal sensor for monitoring grain cleaning losses, however, the signal attenuation time was about 1 s when grains impact the plate, which means that the sensor only can detect 1 grain in 1 s. Its detection frequency was so low that it cannot catch up with the required demand which can cause relative high errors [21]. Gao *et al.* established a FEM model with the LSDYNA software to simulate the grain impact process, and discussed the influences of sensitive plate material on grain impacting process, but no resulting physical sensor designs were applied in the field [22]. Zhou *et al.* developed a grain impact sensor which used a polyvinylidene fluoride (PVDF) piezoelectric-film as sensing element and a signal process circuit was designed. However, they did not take vibration of combine harvester into consideration, and the effect of the designed passive filter circuit was limited [23]. From the above literature, we can know that grain impact sensor mounted in combine harvester mainly focus on pasting a piezoelectric unit on a thin flat sensitive plate. Since the surface of the grain impact sensor usually consists of a rigid sensitive plate, the transient collision signal which is generated after the grain collision decays rapidly in the symmetry plane of the sensitive plate. The transient collision signal is an energy signal which greatly attenuates with time and this greatly affects the detection speed. The shorter the attenuation time, the quicker the vibration system could achieve a steady-state status, and the number of grain impacts that can be distinguished is greatly increased. Grain impact sensors assembled on foreign combine harvesters are mainly used for monitoring grain cleaning losses of wheat, beans and rape. Rice is one of the most important grain crops in China, and currently, both the crop feeding quantity of the combine harvesters and the yield of rice grain—which has wide differences in grain physics properties with wheat, bean and rape—is increasing. Field experiments have showed that current grain impact sensors do not satisfy well the requirements of rice grain harvesting in China, especially in detection speed and sensitivity. On the other hand, due to such a low grain weight, the collision signals were relatively weak, while the vibrations generated by the cleaning sieve, threshing drums, header and engine were so large that they had a significant influence on the accuracy of the sensor, and this resulted in large measurement errors, so there is an urgent need to develop a new sensor which can accurately monitor the grain collisions of rice grains in real time with vibration interference.

To monitor grain separation losses in real time, an indirect method for monitoring grain separation losses on a tangential-axial combine harvester has been presented in this paper. A mathematical monitoring model was derived after detailed comparative data analysis of different feeding quantities. To improve the detection speed and sensitivity of the grain impact piezoelectric sensor, theoretical analysis was carried out from the point of view of structure vibration, and the optimal sensor structural was selected. Finally, a grain impact sensor prototype was assembled on a combine harvester and utilized in field experiments monitoring the mathematics model carried out to verify the validation of the method. Apparently, a proper monitoring mathematical model and a sensor with high accuracy were necessary. The innovation and contributions of this paper can be summarized in the following two aspects: (1) we have established a proper and highly robust mathematical model for monitoring grain separation losses; (2) we have upgraded the sensitivity and detection speed of the grain impact sensor though structure optimization to discriminate between full rice grains and MOG with a relative high accuracy under vibration interference.

## 2. Grain Separation Losses Monitoring Method

**Figure 1.**
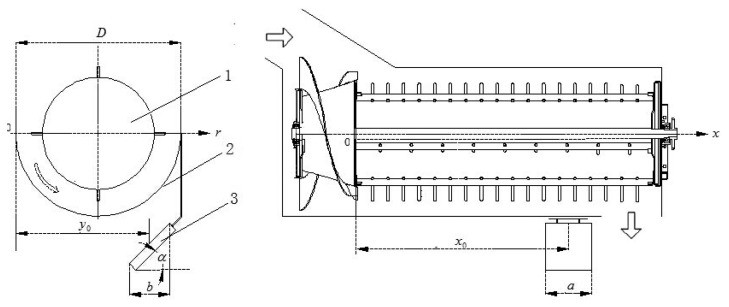
Diagram of grain separation losses monitoring method. 1. Separation rotor; 2. Separation concave; 3. Monitoring sensor.

The separation losses are generally small and involve only a small amount of free grain, which is almost impossible to measure directly when mixed with MOG. To fulfil the task of monitoring grain separation losses in real time, in this paper, we have developed an indirect method which mainly includes the following four steps: (1) draw distribution functions of separated grain in the axial direction of a threshing rotor on a laboratory test-bench and select a proper detection area under the separation concave; (2) establish a mathematical model among relevant variables; (3) develop a grain impact piezoelectric sensor which could discriminate free grains from MOG; (4) fix the sensor on a combine harvester, based on the mathematical monitoring model to calculate grain separation losses in real time. A diagram of the grain separation losses monitoring method is shown in Figure 1.

This method offers interesting opportunities for grain separation losses control systems since it provides a fast estimation of the instantaneous threshability of the crop just two seconds after the crop has entered the header, which can provide a real-time separation losses signal to the control system, and it can avoid malfunctions of the combine harvester in time.

## 3. Development of the Mathematical Monitoring Model

### 3.1. Grain Probability Distribution under the Concave

**Figure 2.**
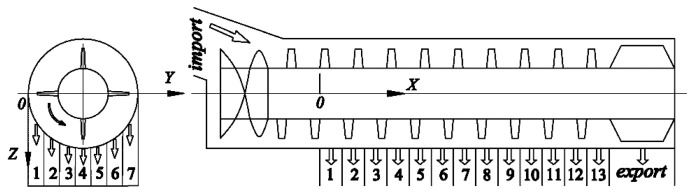
Schematic diagram of coordinate system and location of material's reception boxes.

Miu and Kutzbach have developed a universal mathematical model for grain threshing and separation for a threshing unit [24,25]. On the basis of the previous research works, we extended the mathematical model to be also valid for longitudinal-flow threshing units [26]. Supposing the length of the separation section in a longitudinal-flow rotor was *L* (mm), we set the point above the first receiving box on a longitudinal-flow rotor as the origin of coordinates in an established coordinate system, as shown in Figure 2. The variable *x* was the current position associated with the threshing length.

Suppose *s_a_* was the percentage of free separable grain in the threshing space (%); *s_b_* was the percentage of unthreshed grain in the threshing space (%); therefore, the percentage of grain feeding quantity *q* in the threshing space could be expressed by:

(1) $$q=s_{a}+s_{b}$$

The amount of unthreshed grain *S_n_* is obtained by integrating over the length *x* in the range of [0,*L*], and could be represented through a single exponential function as:

(2) $$S_{n}(x)=s_{b}{\text{e}}^{-\lambda x}$$

According to probability theory, the joint probability density *s_d_*(*x*) of the sum of two independent and steady random variables with the densities *f*(*x*) and *p*(*x*) equals the convolution of their individual probability densities:

(3) $$s_{d}(x)=f(\zeta )*p(x-\zeta )=\int_{0}^{x}f(x)p(x-\zeta )d\zeta$$

Thus, we obtained the joint probability density *s_d_*(*x*):

(4) $$s_{d}(x)=s_{b}\frac{\lambda \beta }{\lambda -\beta }(e^{-\beta x}-e^{-\lambda x})+s_{a}\beta e^{-\beta x}$$

The cumulative distribution function *S_s_*(*x*) of separated grain was found by integrating the probability density function *s_d_*(*x*), and gave:

(5) $$S_{s}(x)=s_{b}\frac{\beta e^{-\lambda x}-\lambda e^{-\beta x}}{\lambda -\beta }+s_{b}+s_{a}(1-e^{-\beta x})$$

Since the material throughput was constant, in the cross-section of threshing space at any current position *x* of separation length, the mass balance can be written as follows:

(6) $$S_{n}(x)+S_{f}(x)+S_{s}(x)=q$$

From Equations (1), (3) and (5), we get the amount of free separable grain *S_f_* as follows:

(7) $$S_{f}(x)=s_{a}e^{-\beta x}-s_{b}(e^{-\lambda x}+\frac{\beta e^{-\lambda x}-\lambda e^{-\beta x}}{\lambda -\beta })$$

At the end of threshing space (*x* = *L*) the free grain became separation losses *V_s_*:

(8) $$V_{s}=S_{f}(L)=s_{a}e^{-\beta L}-s_{b}(e^{-\lambda L}+\frac{\beta e^{-\lambda L}-\lambda e^{-\beta L}}{\lambda -\beta })$$

When the amount of free separable grain was 0 (*s_a_* = 0, *s_b_* = 1), we obtain the grain cumulative distribution function *S_s_*(*x*) and separation losses probability function *V*_s_ of the single axial-flow threshing unit as follows:

(9) $$S_{s}(x)=\frac{1}{\lambda -\beta }\left[\lambda (1-e^{-\beta x})-\beta (1-e^{-\lambda x})\right]$$

(10) $$V_{s}=S_{x}(L)=\frac{\lambda }{\beta -\lambda }(e^{-\beta L}-e^{-\lambda L})$$

### 3.2. Test-Bench Experiments

**Figure 3.**
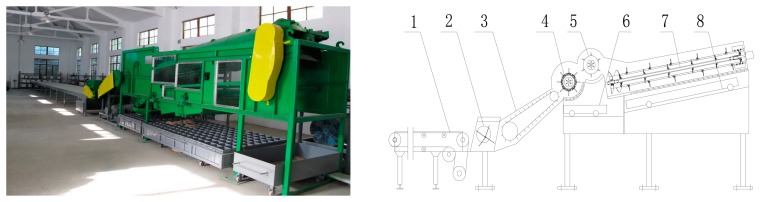
Schematic diagram of the axial threshing-separating-cleaning test-bench. 1. Conveyor belt; 2. Feeding auger; 3. Spout; 4. Tangential-flow threshing unit; 5. Auxiliary feed beater; 6. Tangential-flow reception boxes; 7. Longitudinal-flow threshing unit; 8. Longitudinal-flow reception boxes.

To apply a mathematical model for monitoring grain separation losses, experiments were performed in the laboratory on a longitudinal-axial threshing-separating-cleaning test-bed with tangential feeding [27]. The diameter of the tangential rotor was 590 mm, and its length was 1025 mm. The threshing component of an axial drum with trapezoidal teeth. The diameter of the longitudinal-flow threshing rotor was 500 mm, and its length was 3390 mm, where the length of the separation section *L* = 1950 mm. A schematic diagram of the test-device is illustrated in Figure 3.

**Figure 4.**
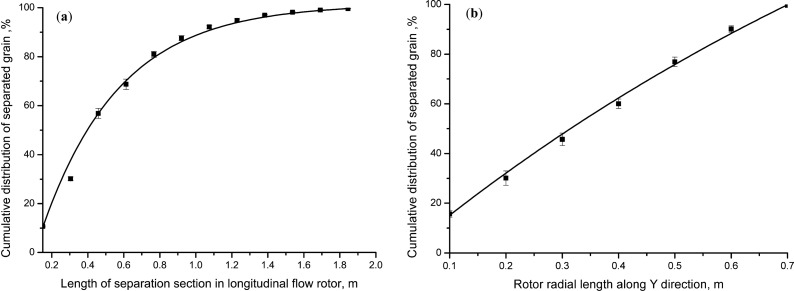
Cumulative distribution of separated grain in the *X* direction and *Y* direction. (**a**) *X* direction; (**b**) *Y* direction

In the experiments, fresh rice was uniformly spread out over an intake chain conveyor (10 m length and 1.2 m wide), then fresh rice was fed into the threshing unit with a constant velocity of 1 m/s; finally, the straw was let out from the grass discharge port at the rear, and the the mixtures fell into the tangential-flow reception boxes or longitudinal-flow reception boxes (13 × 7 matrix, shown in Figure 2; *i* = 1,2…7; *j* = 1,2…13; the size of the box was 150 × 100 mm) through a concave grid. The tangential rotor revolution speed was 800 r/min and the axial rotor revolution speed was 950 r/min. Each experiment was repeated three times to give the averaged values of each box. By using experimental data obtained from experiments with feeding quantities of 6, 7, 8 kg/s, nonlinear fitting carried out and we obtained the results λ = 3.16 m^−1^, β = 4.11 m^−1^, *R*^2^ = 0.9975. The nonlinear fitting result is presented in Figure 4a. For different threshing-separating structures, β was usually distributed in the range of 3.95–5.06 m^−1^ and λ was usually allocated in the range of 3.03–3.95 m^−1^.

Due to the fact the sensor detection speed was constant, a proper grain flow at the sensor mounting position was critical for accurately monitoring grain separation losses. It was turned out that in the leading segment of rotor length direction, the grain rate increases more quickly and the variation of cumulative distribution was larger, while in the end segment (*j* > 12), the amount of separated grains was smaller, but the MOG was massive. There were also distinct grain distribution differences at different radial positions in the range of 10 < *j* < 12. It was found that grains and MOG were mainly distributed in the middle section of the concave, and there were relative fewer amounts of grains and MOG at both ends, so the optimal installation position of the sensor was found to be at the point *i* = 6, *j* = 12 where the mass percentage of grain was most stable, and the variations of other materials were minimum with different feeding quantities.

The distribution probability model of the grain mass ratio under radial direction in the range of *j* = 10–12 can be expressed by:

(11) $$\begin{array}{cc} s_{r}(r)=\frac{r}{D}e^{\chi r(D-r)} & 0\leq r\leq D \end{array}$$

where *r* is radial displace, mm; *D* is diameter of the concave, mm; χ = 1.25–1.62 m^−2^ which obtained by using nonlinear regression analysis, and coefficients of determination *R*^2^ > 0.9920 (χ = 1.32 m^−2^, maximum *R*^2^ = 0.9973); the corresponding nonlinear fitting result is shown in Figure 4b.

### 3.3. Monitoring Mathematical Model

Combining Equations (9), (10), and (11), we obtained a distribution ratio of grains losses in the monitoring area expressed as:

(12) $$f(x_{0},y_{0},a,b)=\frac{{s_{\text{s}}(x)|}_{x_{0}-a/2}^{x_{0}+a/2}\times {s_{r}(r)|}_{y_{0}-b/2}^{y_{0}+b/2}}{V_{s}}$$

where *x*_0_, *y*_0_ was the central position of monitoring area, mm; *a*, *b* was the length and width of the grain losses monitoring sensor, mm. That is, as long as we know *x*_0_, *y*_0_, *a* and *b*, the ratio between the amount of grains in the monitoring area and total grain losses could be calculated according to the Equation (12), and then the sensor measured values converted into total separation losses can monitor the grain separation losses in real time.

## 4. Development of the Grain Impact Sensor

### 4.1. Sensing Element Selection

So far, a measurement method was found for monitoring grain losses by quantifying grain impacts that occur during a fixed interval based on the piezoelectric effect. Therefore, an appropriate piezoelectric material was a key factor to accurately acquire grain collision information. We selected PVDF film (produced by Jinzhou KeXin Electronic Materials Co., Ltd., Jinzhou, China) and YT-5 piezoelectric ceramic (produced by Baoding Sky Ultrasonic Technology Co., Ltd., Baoding, China) as sensing elements, the sensors have been designed respectively, and performance comparison experiments carried out in the laboratory. Property comparison between PVDF film and YT-5 piezoelectric ceramic shown in Table 1.

**Figure 5.**
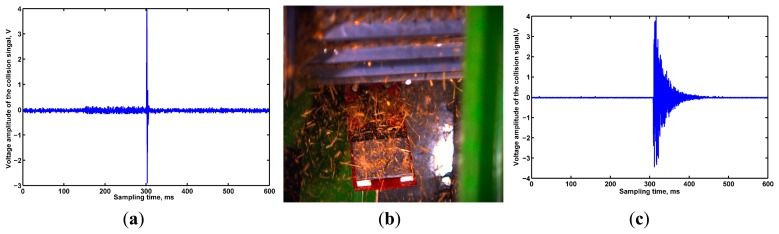
Grain impact sensor performance comparison experiments. (**a**) Signal diagram of grain collision PVDF films; (**b**) Working environment of the PVDF flims; (**c**) Signal diagram of grain collision YT-5L piezoelectric ceramic

It was found that PVDF film has the advantage of high sensitivity to weak impacts [28], and the grain collision signals attenuated rapidly (as shown in Figure 5a), however, in the sensor mounting area, moist powder would be adsorbed on the film's surface which would result in a large area of accumulation (Figure 5b), and this greatly affects the measurement accuracy. Besides, every piece of PVDF film required a unique signal process circuit, which would lead to a large circuit scale and a higher cost.

Piezoelectric ceramic is one of the most widely used sensing materials, and has a high sensitivity to the corresponding changes and could respond to a minimum microvibration, making it especially suited for measuring dynamic changes with a relatively lower cost [29]. By pasting a piezoelectric ceramic in the centre of sensitive plate, grain collision experiments were conducted when four sides of the sensitive plate were fixed, and the corresponding grain collision signal waveforms are shown in Figure 5c. From Figure 5c we could see that the voltage amplitude of the output signal was almost always above 4 V and the overall sensitivity was higher, but the attenuation time was so long that the detection speed could not keep up with the demand. Taking the grain collision signal characteristics, cost, and sensor working circumstances into consideration, we selected YT-5L piezoelectric ceramics as sensing element, a grain impact piezoelectric sensor was designed and structure optimization carried out to improve the detection speed of the sensor.

### 4.2. Characteristics of the Collision Signals

**Figure 6.**
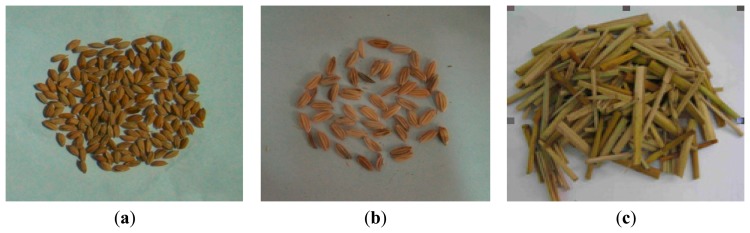
Physical figures of grain and MOG in the mixture (**a**) Full grain; (**b**) blight grain; (**c**) short straw.

The surface of a sensor usually consists of a rigid plane, which discriminates loss grains from MOG based on differences in grain impact force and force rise-time. Since differences in collision force and force rise-time would lead to differences in signal frequency and voltage amplitude, understanding the characteristics of grain collision signal was a decisive step for designing a grain impact sensor. The material ingredients were more complex at the location of the sensor, and besides grain particles (the grain radius ratio general was 1–3 (Xu *et al.* [30]), there were also MOG (mainly composed of long grass (100–300 mm in length), short straw (30–90 mm in length), as well as some light debris), which have a similar physical properties as grains and also have a paramount influence on monitoring accuracy. Pictures of grains and MOG are shown in Figure 6.

**Figure 7.**
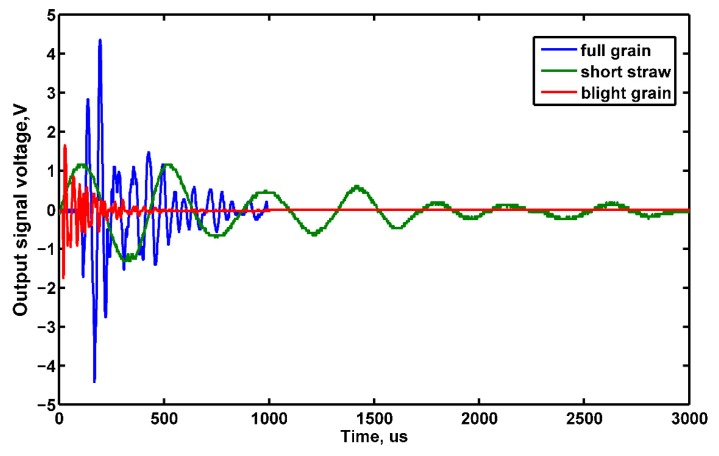
Experimental results of rice grain and MOG impacts with the sensitive plate.

To study the variations of grain collision normal force, collision experiments were carried out in the laboratory with fresh rice and MOG collected in the field (grains with moisture content (MC) about 25.02%; the mass of 1000 full grains was 28.52 g, the mass of 1000 blighted grains was 13.25 g, and short straw MC about 66.43%, the mass of the short straw was distributed in the range of 40–120 mg). The materials were dropped from a height of 350 mm to collide with the sensitive plate. The signals were processed by a charge amplifier and the electric charge converted to a voltage signal before the output voltage signal was recorded by a storage digital oscilloscope (DS01022A, Aglient Technologies, Beijing, China) with a bandwidth of 500 kHz. Owing to its piezoelectric properties and the direction of polarization, the value of the output voltage was proportional to the normal impact force. The averaged values of the experimental results are presented in Figure 7.

Since the horizontal velocity of the material was about zero, the variations of maximum signal voltage *V_out_* and rise time *t_r_* were mainly controlled by the rice shape, orientation and straw length. From Figure 7, it was found that there were distinct differences in *V_out_* and *t_r_* among rice grains, short grains and blighted grains when they impact with the sensitive plate. For rice grains, the maximum *V_out_* was nearly about 4.5 V with *t*_r_ of 25 μs; for short straw, the maximum *V_out_* was nearly about 1.4 V with *t_r_* of 100 μs; and for blighted grain, the maximum *V_out_* was nearly about 1.7 V with *t_r_* of 12 μs.

According to the differences in *V_out_* and *t_r_* which lead to corresponding differences in signal frequency and voltage amplitude, a signal process circuit was designed. The circuit mainly consisted of a band-pass filter circuit with a corner frequency of 5–20 kHz and a comparator circuit. Because of the stochastic collision nature and initial velocity of the grains when they make contact with the sensitive plate, the generated signal peak voltage may be a positive or negative value. In order to acquire the peak voltage accurately, an absolute value amplifier consisting of a precision detector and an adder were developed. To adjust the sensitivity of the sensor according to current condition, a voltage comparator circuit was designed which also can inhibit the vibration interference by changing the threshold value of the comparator circuit. Finally, the output signal was conveyed into a monostable trigger which was constituted by a NE555 counter to get a standard square wave signal. The standard square wave signal can be received by the MCU and the width of the square wave can be adjusted.

### 4.3. Required Detection Speed of the Sensor

Detection speed is an essential parameter to evaluate the performance of the sensor, and the grain flow rate at the sensor mounting position was also required for testing the sensor's ability. To select an appropriate grain flow for testing the detection accuracy of the sensor, threshing experiments were carried out on the threshing-separation test bench (shown in Figure 1) with feeding quantities of 4.0–8.0 kg/s, and we thus obtained the grain flow rate at the sensor installation position. The experimental results are shown in Table 2.

From Table 2, we can see that as the quantity fed increases, the grain flow rate in the monitoring area increases gradually. The maximum grain flow rate was up to 120 grains per second, which means that the attenuation time of the collision signal should be less than 8 ms. As seen from the grain collision original signal waveform shown in Figure 5c, the attenuation time was so long that the detection speed of the sensor could not be guaranteed. Therefore, to accommodate the required speed in a huge range, we must take some actions to accelerate the detection speed.

### 4.4. Structure Optimization of the Sensor

#### 4.4.1. Structure Parameters Selection of the Sensor

According to Passion-Kirchhoff flat theory, the vibrations generated by grain collisions with a sensitive plate satisfy the equation:

(13) $$D\left[\frac{\partial^{4}w}{\partial x^{4}}+2\frac{\partial^{4}w}{\partial x^{2}\partial y^{2}}+\frac{\partial^{4}w}{\partial y^{4}}\right]+\rho h\frac{\partial^{2}w}{\partial t^{2}}=F(x,y,t)$$

When four sides of the plate are fixed, the vibration modes of the sensitive plate could be articulated as follows:

(14) $$w(x,y)=C(1+\cos\frac{\pi x}{a})(1+\cos\frac{\pi y}{b})$$

The natural frequencies of the plate could be calculated as follows according to Green's formula:

(15) $$T_{\max}=\frac{1}{2}\iint m\omega^{2}W^{2}\text{dxdy}$$

(16) $$U_{\max}=\frac{D}{2}\iint \left\{{\left(\nabla^{2}w\right)}^{2}-2(1-\nu )\left[\frac{\partial^{2}w}{\partial x^{2}}\frac{\partial^{2}w}{\partial y^{2}}-{\left(\frac{\partial^{2}w}{\partial x\partial y}\right)}^{2}\right]\right\}\text{dxdy}$$

(17) $$U_{\max}=T_{\max}$$

(18) $$\omega_{1}=\frac{4\pi^{2}}{3a^{2}}\sqrt{3+\frac{2a^{2}}{b^{2}}+\frac{3a^{4}}{b^{4}}}\sqrt{\frac{D}{m}}$$

where *C* was a constant value, *T*_max_ was the maximum kinetic energy, *U*_max_ was the maximum potential energy, $\nabla^{2}=\frac{\partial^{2}}{\partial x^{2}}+\frac{\partial^{2}}{\partial y^{2}}$ was called biharmonic operator, $D=\frac{Eh^{3}}{12(1-\mu^{2})}$ was called the flexural rigidity of the plate, *w*(*x*, *y*) was the deflection of the middle plane in the *z* direction, *μ* Poisson's ratio, *E* was Yong's modulus of elasticity, *ρ* was density of the plate, *h* was thickness of plate, *a* was length of the plate, *b* was width of the plate, *F* was excited force, ω_1_ was the first order circular frequency.

**Figure 8.**
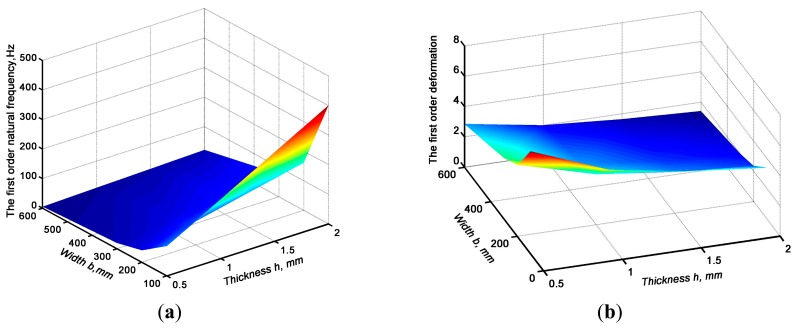
Effect of structure on the first order natural frequency and relative deformation ratio. (**a**) effect of width and thickness on the first natural frequency; (**b**) effect of width and thickness on the first order deformation

From Equations (14) and (18), it is shown that the natural frequencies and vibration modes of the sensitive plate mainly depend on *h*, *a* and *b* under the condition that *E*, *ρ* and *μ* are fixed. Therefore, selection of the sensitive plate geometry was a key technology for improving the performance of the sensor. Relevant research indicated that low levels of vibration had a larger effect on the dynamic performance of the structure: the higher of the first-order natural frequency, the quicker the vibration system could reach a steady-state status; the larger the vibration displacement, the greater the deformation of the piezoelectric element that occurred was, and a greater amount of charge would be generated, so the sensor sensitivity was also higher. To obtain variations of the first-order natural frequency and relative deformation rate under different structural forms, modal analysis was carried out though ANSYS software (ANSYS, Inc., Canonsburg, PA, USA, 2011). In modal analysis, we utilized the Shell 63 element as the calculation unit, and sensitive plate material was 304 stainless steel with *μ* = 0.3, *E* = 210 GPa, and *ρ* = 7,850 kg/m^3^, *a* = 120 mm, *b* = 100 mm, 200 mm, 300 mm and 600 mm, *h* = 0.5 mm, 1.0 mm, 1.5 m m and 2.0 mm; the calculated results are shown in Figure 8.

From Figure 8 we can see that with the increase in thickness *h*, the first-order natural frequency increased, and relative deformation rate decreased, but along with increase of width *b*, the first-order natural frequency and the relative deformation rate all decreased monotonously. In the range of width *b* > 300 mm, thickness *h* > 1.0 mm, with increase of thickness *h*, the first-order natural frequency and the relative deformation rate increased, but the growth rate was relatively small, and showed a trend of gradually stabilizing at a constant value. Taking the sensitivity and attenuation time into consideration, a sensitive plate which width *b* = 200 mm, thickness *h* = 1.50 mm was selected, which might be the optimized size for the sensitive plate.

**Figure 9.**
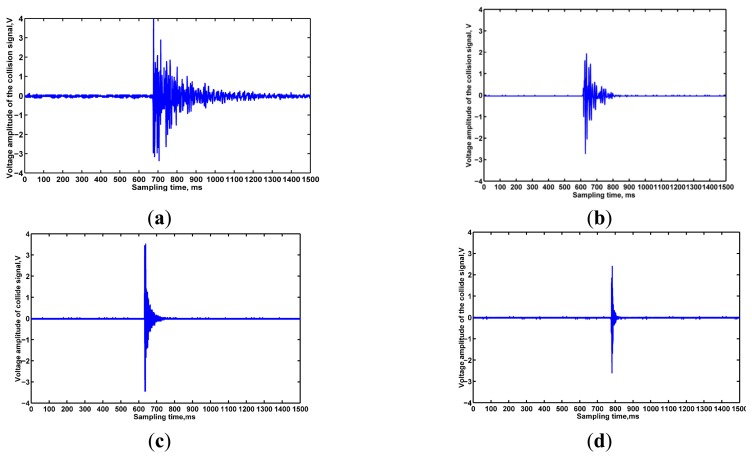
Grain collision signal waveform on different structures. (**a**) *h* = 0.5, *b* = 400; (**b**) *h* = 2.0, *b* = 400; (**c**) *h* = 1.0, *b* = 200; (**d**) *h* = 2.0, *b* = 200.

To verify the ANSYS software simulation results and analyze the influence of structure on detection performance intuitive contrast grain collision experiments were performed in the laboratory. The experimental process was the same as the method mentioned above and the resulting impact signals using four dipfferent structures are shown in Figure 9. Figure 9 shows that, when the sensitive plate width *b* = 400 mm, the first-order natural frequency of the sensitive plate was increased as the thickness *h* increased from 0.5 mm to 2.0 m,, while the relative deformation rate monotonously decreased. Accordingly, the collision signal attenuation time and voltage amplitude both decreased. Similarly, when the sensitive plate length *b* = 200 mm, the first-order natural frequency of the sensitive plate increased and tended to a constant range, while the relative deformation rate monotonously decreased with increases of thickness *h* from 1.0 mm to 2.0 mm. The collision signal attenuation time has a rare decline and the voltage amplitude is decreased to a large extent, which would affect the sensitivity of the sensor. Further grain collision experiments results verified that the experimental results were basically consistent with the theoretical analysis results. The higher the first natural frequency, the shorter the signal attenuation time was; the higher the relative deformation rate, the higher the sensor's overall sensitivity. Taking the monitoring area, sensor performance under different structures, and economy as considerations, a sensitive plate with length *a* = 120 mm, width *b* = 180 mm, thickness *h* = 1.0 mm was selected as the optimal structure.

### 4.4.2. Partially Constrained Damping Design of the Sensor

**Figure 10.**
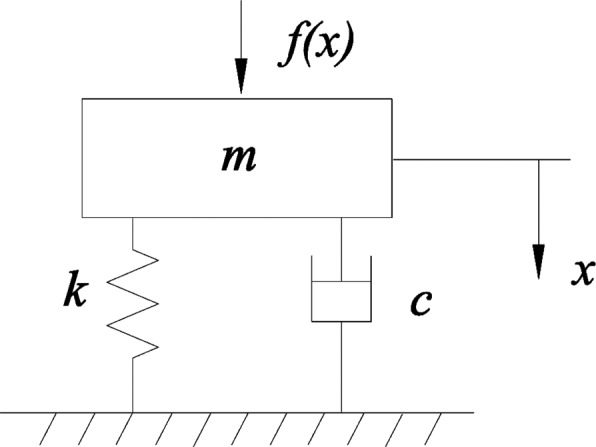
Simplified dynamics model of the grain collision process.

From above explanation we know that the current detection speed could not meet the demands if the quantity fed was larger than 7 kg/s, so the sensor structure should be optimized further. Relevant research has indicated that the damping ratio of the sensor has a paramount influence on grain collision response [30,31]. To exploit their relationship, the grain collision process was simplified to a dynamic model, shown in Figure 10, which kinetic equation could be expressed as:

(19) $$\overset{..}{y}(t)+2\zeta \omega_{n}\dot{y}(t)+{\omega_{n}}^{2}y(t)=pf(t)$$

where, ω*_n_* was the natural frequency, *ζ* was the viscous damping factor, *f*(*t*) was the input signal, *y*(*t*) was the output signal, *p* was the input gain.

Because the sensor damping ratio was very small, the sensor could be seen approximately as a second-order system. Due to the very short collision time, the collision response between grain and sensitive plate could be seen as a unit impulse response to a second-order system, which collision response function could be expressed as follows:

(20) $$X(t)=\{\begin{array}{ll} \frac{\hat{F}}{m\omega_{d}}e^{-\xi \omega_{d}t}\sin\omega_{d}t & \left(t>0\right)\\ 0 & \left(t\leq 0\right) \end{array}$$

where, *F̂* was the impulse force, *N*; *ω_n_* was natural frequency of undammed oscillation, rad/s; damping ratio $\zeta =\frac{c}{c_{c}}$, *c_c_* was critical damping coefficient and *cc* = 2(*mk*)^1/2^, *c* was damping coefficient; ω*_d_* was angular frequency of damped vibration, $\omega_{d}=\sqrt{1-\zeta^{2}}\omega_{n}$, rad/s, ω*_n_* = (*k*/*m*)^1/2^.

Since the damping ratio of the second-order system was very small, from Equation (20) we can know that the system became a sharp vibration resonance system, which would transform the input signal into an approximate harmonic oscillation signal form. The attenuation time of the output signal would become longer after envelope detection, which led to the detecting speed and measurement accuracy declining significantly. In addition, when ζ was relatively small, it also leads to decreased stability of the sensor to some degree. To analyse in depth the relationship between vibration system damping and grain collision response from the perspective of structure vibration, the frequency characteristic *G*(*j*ω) of the vibrated displacement *x* for excitation force *f*(*t*) was obtained through the Laplace transform:

(21) $$G(j\omega )=\frac{k+jc\omega }{k-m\omega^{2}+jc\omega }$$

Separating the real and imaginary parts of *G*(*j*ω), we obtain the analytical expression of the amplitude-frequency characteristic for the vibration system in polar coordinates as:

(22) $$G(j\omega )=R(\omega )e^{j\theta (\omega )}$$

where *R*(ω) was called the amplitude-frequency characteristics in polar coordinates and can be expressed as:

(23) $$R(\omega )=\frac{1}{{\left[{\left(k-m\omega^{2}\right)}^{2}+c^{2}\omega^{2}\right]}^{1/2}}$$

Then the dynamic magnification coefficient of the vibration system was calculated as:

(24) $$A(g)=\frac{1}{{\left[{\left(1-g^{2}\right)}^{2}+4\zeta^{2}g^{2}\right]}^{\overset{1}{\;}/\underset{2}{\;}}}$$

where *g*=ω/ω_*n*, *ω*_ is the excitation frequency.

From Equation (24) it could be observed that a vibration resonance would happen when the sensitive plate damping ratio *ζ* was very small, and when the dynamic magnification coefficient *A*(*g*) was very large, the vibration system would amplify interference signals easily. On the one hand, due to the limited capacity to attenuate the vibration, the attenuation time of grain collision signals would be longer; on the other hand, when the voltage amplitude of interference signal was amplified more than the threshold voltage of the voltage comparator, this would cause large measurement errors by mistake. Therefore, an appropriate damping ratio *ζ* was the basis for detecting a signal with a short of attenuation time.

Increasing damping of the vibration system was an effective way to rapidly attenuate vibrations. Additional damping was achieved by adding a viscoelastic material layer with high resistance to a vibration system to increase the system's damping capacity and consume the vibration energy in time. The new viscous damping ratio could be expressed as:

(25) $$\zeta^{\prime }=(1+\nu )\zeta$$

where, constant value $\nu =\frac{c_{1}}{c}$, *c*_1_ was the additional damping coefficient.

**Figure 11.**
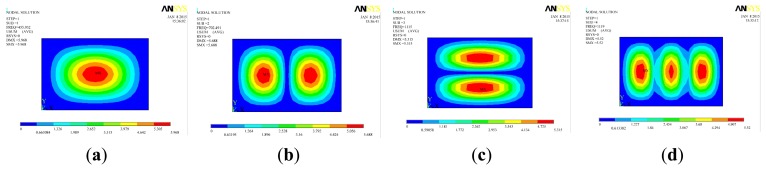
The first 4 order modal shape contour of sensitive plate (**a**) modal 1; (**b**) modal 2; (**c**) modal 3; (**d**) modal 4.

If *c*_1_ ≫ *c*, then we get ζ′ ≫ ζ, from Equation (24) and it can be seen that the dynamic magnification coefficient *A* (g) was significantly decreased, so that the harmonic vibration can be rapidly decayed. A constrained damping layer which is based on shear deformation of the viscoelastic material between the substrates consumes vibration energy. The location of the constrained damping layer has a greatly effect on structural vibration attenuation. The vibration damping effect was limited when the constrained damping layer is applied in a small deformation place, so the constrained damping layer should cover the place with larger structural deformation, which would achieve a better vibration damping effect, as well as result in a small increase of the total weight of the sensor. It was known that vibration modes of the sensitive plate determined on condition of *h*, *a*, *b* and material of the sensitive plate were fixed. To obtain the relative deformation rate of the plate, modal analysis utilizing Shell 63 element as calculation unit was carried out with the ANSYS software. Relevant research has indicated that low-level vibration has a larger effect on the dynamic performance of the structure, so we selected the first four orders of vibration modes and the calculated results are shown in Figure 11.

Figure 11 indicates that the modal deformation of the sensitive plate was very small in the place close to a fixed side, while the mode deformation was larger in the centre of the sensitive plate. By mounting the sensitive element in the center of the sensitive plate, the sensor would have a relative higher sensitivity; and the sensor would achieve a relatively high detection speed if the viscoelastic material were pasted in the larger modal deformation place.

**Figure 12.**
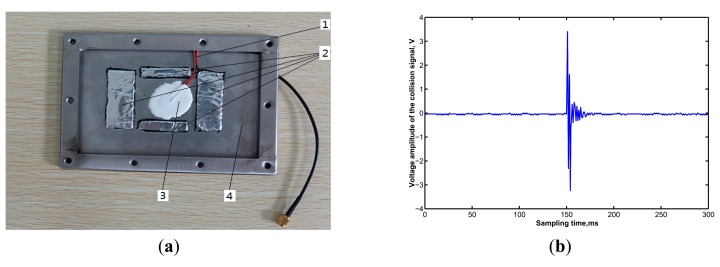
Collision signal waveform when damping layer laying on the optimal position. 1. Signal wire; 2. Viscoelastic material layer; 3. Piezoelectric ceramic; 4. Sensitive plate.

To verify the theoretical analysis results, grain collision experiments were performed in the laboratory. It was found that when the viscoelastic layer, which material was isobutylene isoprene rubber and with a thickness of 2 mm, was laid out on different places of the sensitive plate, the attenuation time of the grain collision signals displayed great differences. On the basis of grain collision experiments, we determined an optimal position to lay the damping layer, as shown in Figure 12a. The grain collision signal, shown in Figure 12b, indicated that the attenuation time of the grain collision signal was shortened to about 3 ms, which showed that the sensor could identify more than 300 grains within 1 s in theory; and the sensor has a relative higher sensitivity with a maximum signal amplitude of about 4 V. Compared with the sensors mentioned above installed on most commercial harvesters and sensors developed by Ni *et al.* [21]. which attenuation time was about 1 s, and the maximum signal amplitude was less than 1.2 V, the sensitivity and detection speed of the sensor were both greatly improved.

#### 4.4.3. Sensor Performance Tests

**Figure 13.**
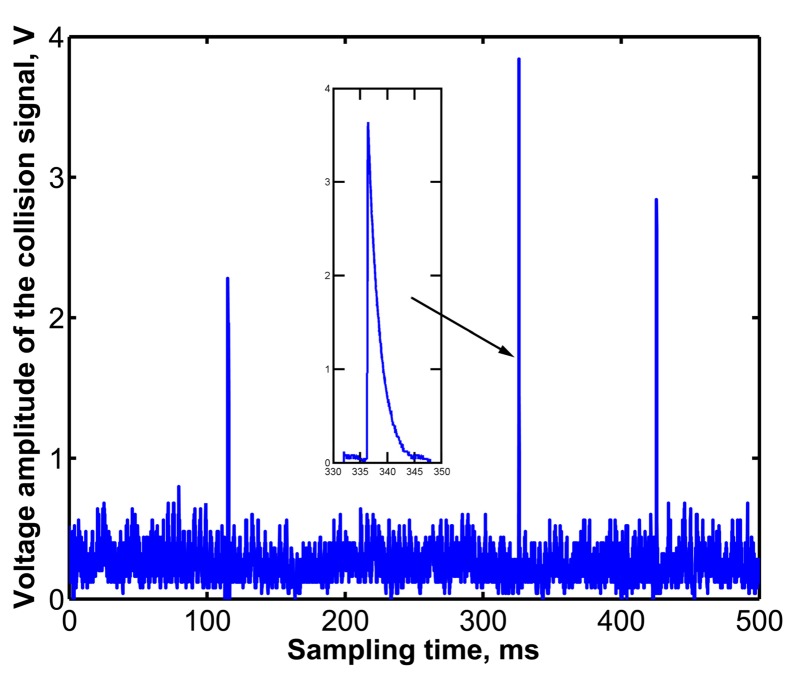
Output signal's waveform of grain collision under vibration interference.

Vibration also has a significant influence on the monitoring accuracy of the sensor, so to avoid the influence of vibration, a passive vibration isolation structure [32,33] had been designed which involved pasting an isolating material with a stiffness *k* = 500 N/m and thickness of 3 mm between the sensitive plate and the sensor base using glue, and then, installing the sensor on a combine harvester. The combine harvester and the working parts of the combine harvester were started-up in a normal working state, and a the digital oscilloscope (DS01022A, Aglient Technologies, Beijing, China) was used to record the output signal of the sensor, with sampling frequency 500 kHz. Output signals under vibration interference are shown in Figure 13.

From Figure 13 we can see that the rubber with stiffness of *k* = 500 N/m has a good effect on isolating vibrations and was therefore a suitable material for vibration isolation. The output voltage amplitude of full rice grain collision signals was generally in the 2–4 V range, while for MOGs, the voltage amplitude of the output signal was always below 1.0 V. Therefore, by setting an appropriate threshold voltage of the comparator circuit we could almost eliminate the influence of MOGs and vibration interference on sensor monitoring accuracy.

## 5. Field Experiments

**Figure 14.**
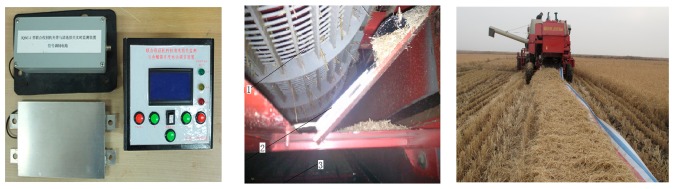
Installed position of the sensor on a combine harvester and experiments. 1. Separation concave; 2. Monitoring sensor; 3. Cleaning sieve.

A prototype grain impact piezoelectric sensor was assembled on a tangential-longitudinal axial combine harvester (Model: 4LQZ-6 Foton Lovol International Heavy Industry Co., Ltd., Weifang, China) as shown in Figure 14. Its header width was 4.75 m and had the same parameters as the test-bench shown in Figure 2. Field experiments were carried out on Baoquanling Farm, Heilongjiang Province, China. In the sensor mounting area, we substituted *x_0_* = 1.8 m, *y_0_* = 0.6 m, *a* = 180 mm, *b* = 120 mm, *χ* = 1.32 m^−2^, *λ* = 3.16 m^−1^, *β* = 4.11 m^−1^ into Equation (12), and got a distribution ratio *f* = 1/1.37.

Before the formal experiments, the threshold voltage of the signal processing circuit was adjusted until the value displayed on the LCD screen was about 1–2 grains when the combine harvester worked in a normal state, which indicated that the system could overcome vibration interferences. We input the value of *f* into the display instrument, and the harvesting distance was 15 m. An oil skin was used to collect all the mixed material at the outlet of the sieve, then the full grains were filtered out from material-other-than-grain (MOG) manually, weighed and the separation losses calculated. Based on the sensor counted grain numbers and monitoring mathematical model, the total grain separation losses were calculated. Table 3 gives the error analysis of the results acquired by the sensor and manually under the same operating parameters.

From Table 3 it can be seen that the relative measurement error was <4.63%, compared with the results of Tang *et al.* who established a threshing-separating matrix equation to calculate grain separation losses on the tangential-longitudinal-axial combine harvester and got a relative calculated error in the range of 4.82%–5.87% which indicated that the developed rice grain separation losses monitoring system worked well. Due to the complexity of the threshing process, the grain number counted by the sensor was not a constant value. The reasons for this are the following aspects: (1) dropped grains would collide with the sensitive plate again because of the interactions among grains and MOG, after that different lateral vibration displacement occurred as different parts of grain contact the sensitive plate, and then varied amplitudes of the voltage signal would be generated. When the signal voltage amplitude was less than the threshold voltage of the voltage comparator circuit, the grain impact piezoelectric sensor may cause a leakage and this leads to a substantial measurement error. (2) Since short straws generally have knots, which have similar physical and mechanical properties as full grains, the sensor would cause a measurement error when a knot collides with the sensor. (3) Besides, due to the rugged ground, the combine harvester would produce severe turbulence during the working operation in the field, which would cause a failure of the isolation device, resulting in a large measurement error. (4) In addition, when the instantaneous quantity fed to the combine harvester was too large, lots of outputs would be produced beneath the rotor in a very short of time, and if the amount of outputs was beyond the sensor's monitoring capacity this also would lead to a large measurement error.

## 6. Conclusions

To monitor separation losses in real time, a method for monitoring grain separation losses was proposed in this paper. A distribution probability model of the separated grains along the radial and axial direction of the longitudinal-flow rotor derived after detailed comparative analysis of different feeding quantities, and a mathematical monitoring model were established. According to differences in voltage amplitude and rise-time which would lead to differences in signal frequency and voltage amplitude a signal processing circuit was developed which mainly consists of a band-pass filter circuit with the corner frequency of 5–20 kHz and a comparator circuit. The sensor structure has a great influence on the sensor's performance, so in order to speed up the detection speed of the sensor, a theoretical analysis was carried out at the point of vibration. It was found that the attenuation time of the grain collision signals was shortened to about 3 ms and the maximum signal amplitude was nearly about 4 V when the sensitive plate treated with a partially constrained damping layer in places undergoing larger deformations under the condition of length *a* = 120 mm, width *b* = 180 mm, thickness *h* = 1.0 mm. Compared with other sensors equipped on most commercial harvesters and sensors developed by Ni *et al.* [21]. which attenuation time was about 1 s, and the maximum signal amplitude was less than 1.2 V, the sensor detection speed was greatly improved. Finally, to avoid the effect of vibration on measurement accuracy, a passive vibration isolation structure was designed. By assembling the sensor on a combine harvester and utilizing the mathematical monitoring model developed based on laboratory test-bench experiment results, field tests were carried out. The test results indicated that the measurement error was less than 4.63%. Compared with Tang *et al.* who established a threshing-separating matrix equation to calculate grain separation losses on the tangential-longitudinal-axial combine harvester and got a relative calculated error in the range of 4.82%–5.87%, the measurement error of method presented in this paper was greatly decreased, which indicated that the developed rice grain separation loss monitoring system worked well.

This method offers interesting opportunities for grain separation losses control systems since it provides a fast estimation of the instantaneous threshability of the crop just 2 s after the crop has entered the header, but 6 s before the flow variation reaches the exhaust port, which can provide a real-time separation losses signal to the control system and can avoid combine harvester malfunctions in time.

## Figures and Tables

**Table 1.** Property comparison between PVDF film and YT-5 piezoelectric ceramic.

**Parameters**		**PVDF**	**YT-5**
Electromechanical coupling coefficient	*K*_33_	0.14	0.71
Piezoelectric constant PC/N	*d*_31_	23	270
	*d*_33_	21	550
Relative permittivity εTr_3_		10	7.0
Curie temperature/C *T*_c_		80	280
Mechanical quality factor *Q*_m_		0.018	70
Density g/cm^3^ ρ*_m_*		1.78	7.6

**Table 2.** Grain flow rate in sensor monitoring regional under different feeding quantity.

Quantity fed /(kg/s)	4.0	4.5	5.0	5.5	6.0	6.5	7.0	7.5	8.0
Flow rate/(grains/s)	20	36	55	61	75	82	96	108	120

**Table 3.** Error analysis of separation losses obtained by sensor compared to manual measurement.

**Tests No.**		**Forward Speed m/s**	**Separation Losses**				

			**Sensor**		**Manual**		**Relative Error/%**

			**Total Amount/g**	**Ratio/%**	**Total Mass/g**	**Ratio/%**	
1#	1	0.8	198.5	0.260	202.4	0.265	1.96
	2	1.0	224.6	0.294	230.8	0.302	2.76
	3	1.2	244.2	0.320	251.6	0.329	3.03

2#	1	0.8	165.2	0.293	170.4	0.303	3.30
	2	1.0	199.2	0.354	206.8	0.368	3.80
	3	1.2	230.6	0.410	220.4	0.392	4.63
